# Detection of mutational patterns in cell‐free DNA of colorectal cancer by custom amplicon sequencing

**DOI:** 10.1002/1878-0261.12539

**Published:** 2019-07-19

**Authors:** Simon Herrmann, Tianzuo Zhan, Johannes Betge, Benedikt Rauscher, Sebastian Belle, Tobias Gutting, Nadine Schulte, Ralf Jesenofsky, Nicolai Härtel, Timo Gaiser, Ralf‐Dieter Hofheinz, Matthias P. Ebert, Michael Boutros

**Affiliations:** ^1^ Division Signaling and Functional Genomics German Cancer Research Center (DKFZ) Heidelberg Germany; ^2^ Department Cell and Molecular Biology Faculty of Medicine Mannheim Heidelberg University Germany; ^3^ Department of Internal Medicine II Medical Faculty Mannheim Heidelberg University Mannheim Germany; ^4^ Medical Faculty Mannheim Institute of Pathology Heidelberg University Mannheim Germany; ^5^ Medical Faculty Mannheim Interdisciplinary Tumor Centre Heidelberg University Mannheim Germany; ^6^ German Cancer Consortium (DKTK) Heidelberg Germany

**Keywords:** cfDNA, colorectal cancer, liquid biopsy, next‐generation sequencing

## Abstract

Monitoring the mutational patterns of solid tumors during cancer therapy is a major challenge in oncology. Analysis of mutations in cell‐free (cf) DNA offers a noninvasive approach to detect mutations that may be prognostic for disease survival or predictive for primary or secondary drug resistance. A main challenge for the application of cfDNA as a diagnostic tool is the diverse mutational landscape of cancer. Here, we developed a flexible end‐to‐end experimental and bioinformatic workflow to analyze mutations in cfDNA using custom amplicon sequencing. Our approach relies on open‐software tools to select primers suitable for multiplex PCR using minimal cfDNA as input. In addition, we developed a robust linear model to identify specific genetic alterations from sequencing data of cfDNA. We used our workflow to design a custom amplicon panel suitable for detection of hotspot mutations relevant for colorectal cancer and analyzed mutations in serial cfDNA samples from a pilot cohort of 34 patients with advanced colorectal cancer. Using our method, we could detect recurrent and patient‐specific mutational patterns in the majority of patients. Furthermore, we show that dynamic changes of mutant allele frequencies in cfDNA correlate well with disease progression. Finally, we demonstrate that sequencing of cfDNA can reveal mechanisms of resistance to anti‐Epidermal Growth Factor Receptor(EGFR) antibody treatment. Thus, our approach offers a simple and highly customizable method to explore genetic alterations in cfDNA.

**Abbreviations**
CEA: carcinoembryonic antigen
cfDNA: cell‐free DNA
CRC: colorectal cancer
ctDNA: circulating tumor DNA
EGFR: Epidermal Growth Factor Receptor
LDH: lactate dehydrogenase
UMI: unique molecular identifiers
VEGF: Vascular Endothelial Growth Factor

## 1 Introduction

Although survival rates have been increasing over the last decades, the prognosis of patients with advanced stages of gastrointestinal cancer is persistently poor (Jemal *et al*., 2011). Major challenges include tumor heterogeneity and the clonal expansion of resistant clones during the course of treatment, resulting in cancer progression or relapse (Alizadeh *et al*., 2015). Monitoring the dynamics of cancer evolution during the course of a treatment may help to early identify the development of drug resistance. However, in patients with advanced disease, obtaining serial tumor biopsies to study tissue‐based biomarkers is not feasible in the majority of cases. Most biopsy techniques bear risks due to their invasiveness and are not suitable for morbid patients (Vanderlaan *et al*., 2014). Besides, a tissue biopsy only represents a small section of a heterogenous and disseminated disease, ignoring cancer biology at other (metastatic) sites. Hence, it may result in sampling errors due to branched evolution of metastatic sites and intratumoral heterogeneity (Burrell *et al*., 2013; Gerlinger *et al*., 2012).

It is known for decades that solid tumors can release single tumor cells (Alix‐Panabières and Pantel, 2013; Maheswaran and Haber, 2010) or nucleic acids into the bloodstream, including DNA, mRNA, and miRNA (Schwarzenbach *et al*., 2011; Stroun *et al*., 2006). Advances in DNA sequencing opened up opportunities to analyze the genetic composition of this circulating tumor DNA (ctDNA) (Wan *et al*., 2017). In fact, large‐scale sequencing projects of ctDNA showed that genetic alterations present in primary tumors can also be found in ctDNA (Strickler *et al*., 2018; Zill *et al*., 2018). Furthermore, serial ctDNA analysis proves to be a powerful method to longitudinally monitor therapy response and the emergence of resistant clones (Bettegowda *et al*., 2014; Siravegna *et al*., 2015; Thierry *et al*., 2017). Several methods have been developed to detect mutations in ctDNA, including BEAMing technologies (Diaz *et al*., 2012; Haselmann *et al*., 2018; Janku *et al*., 2015), panel sequencing of cancer‐associated genes (Strickler *et al*., 2018; Zill *et al*., 2018), or targeted amplicon sequencing (Lebofsky *et al*., 2015; Tie *et al*., 2016). While every method offers advantages, a major challenge to all technologies is the divergent mutational landscape of cancer. While some mutations in key oncogenes occur at high frequency across many tumors, most genetic alterations are tissue‐specific or even highly variable between cancers of the same tissue origin (Lanman *et al*., 2015). Current FDA‐approved diagnostic tests for cell‐free DNA (cfDNA) cover mutational hotspots of large predefined panels of frequently altered genes in cancer (Lanman *et al*., 2015). While this approach is suitable for application in a broad spectrum of cancers, it is not designed to detect potentially relevant mutations that occur only in specific tumors, such as RNF43 in colorectal (CRC) and pancreatic cancer (Steinhart *et al*., 2017). Thus, complementary tests are required that can be used to explore a custom‐defined set of genetic alterations in cfDNA that are not covered by commercially available tests. These tests are valuable to correlate the occurrence of rare mutations with disease outcome or drug resistance.

Here, we present an end‐to‐end experimental and bioinformatic workflow that can be used to analyze a custom set of genetic alterations in cfDNA. We designed an amplicon panel that is tailored to the genetic landscape of CRC, covering mutations in oncogenes and tumor suppressors as well as functionally relevant mutations in genes that are less frequently altered. We performed amplicon sequencing with our custom panel in serial cfDNA samples from a pilot cohort of 34 patients with metastatic CRC. Using this approach, we detected patient‐specific mutational patterns that reoccurred in serial samples. We also show that the mutant allele frequencies of genetic alterations in cfDNA increase with disease progression.

## 2 Materials and methods

### 2.1 Study design and clinical databank

Patients with gastrointestinal cancers across all tumor stages were included in the study. Blood was collected before start of anticancer treatment and every 3–6 months during radiologic restaging or if patients were hospitalized. Simultaneously, the following blood markers were determined: cell counts of erythrocytes, leukocytes, and thrombocytes; AST; ALT; GGT; ALP; bilirubin; albumin; creatinine; INR; LDH; CRP; and the serum tumor markers CEA, CA19‐9, and AFP. Clinical characteristics were collected in a prospective database.

Written informed consent was obtained from all patients, and the study was approved by the local ethics board (Ethikkommission II, Medical Faculty Mannheim, Heidelberg University, identifier 2013‐640N‐MA). The study design is in accordance with the standards proposed by the Declaration of Helsinki.

### 2.2 Sample collection and plasma DNA extraction

Two 10‐mL K_3_EDTA tubes were taken during regular blood sampling and before administration of chemotherapy. Blood samples were immediately stored at 4 °C and processed within 16 h to minimize release of genomic DNA from nontumorous sources (Norton *et al*., 2013; Risberg *et al*., 2018; Wong *et al*., 2013). For isolation of blood plasma, K_3_EDTA tubes were centrifuged at 1900 ***g*** for 10 min at 4 °C. Afterwards, supernatants were centrifuged at 16 000 ***g*** for 10 min at 4 °C to remove remaining blood cells. Plasma was harvested without mobilizing the cell pellet and stored at −80 °C until needed. For cfDNA isolation, 1–4 mL of frozen plasma was used. Extraction of DNA was performed using the QIAamp DNA Blood Mini Kit (Qiagen, Hilden, Germany). DNA was dissolved in 50–70 μL nuclease‐free water and stored at −20 °C for further use. The amount of DNA was quantified with qubit 2.0 (Life Technologies, Carlsbad, CA, USA) using the high‐sensitivity assay according to the manufacturer's protocol.

### 2.3 Design of amplicon primers and multiplex reaction

Positions of frequently mutated hotspots in CRC‐associated genes were obtained from the COSMIC database (Forbes *et al*., 2015). In addition, frequently mutated loci in genes that regulate the metabolism of 5‐fluorouracil (*DPYD*,* TYMS*,* TYMP*,* UPP1*) and mismatch repair (*MLH1*,* MLH3*,* MSH6*,* PMS2*) were selected. Nucleotide sequences surrounding the genetic alteration of interest were obtained from the NCBI database with the genome assembly GRCH38 as a reference. Specific primers for regions that cover the selected mutations were designed with Primer3 (Untergasser *et al*., 2012) with the following settings: primer length of 19–27 bp, GC ratio of 30–63%, and melting temperature of 55–61.5 °C. The universal adapter sequence TCCCTACACGACGCTCTTCCGATCT was added to the 5′ end of each forward primer. To each reverse primer, the sequence AGTTCAGACGTGTGCTCTTCCGATC was added to the 5′ end. The amplicon length varied between 100 and 175 bp. Characteristics and sequences of all primers can be found in Table S1. To develop a multiplex PCR assay, we used the software tool multiplx 2.1 (Kaplinski and Remm, 2015) to identify appropriate primer combinations. To this end, we set the concentration of monovalent salts to 50 mm and the concentration of Mg^2+^ to 1.5 mm. All five possible primer interaction scores that might affect primer compatibility were calculated. To group the 43 primer pairs for multiplex PCR, we used the stringency value ‘normal’. The best combination resulted in six different pools with 5–9 primer pairs.

### 2.4 Nested PCR protocol and library preparation

We developed a custom, nested PCR protocol suited for multiplex reactions. For the first PCR step, at least 500 pg of cfDNA was used per multiplexed reaction. In all cases, we used Q5 Hot Start Polymerase (NEB, Ipswich, MA, USA) to minimize amplification errors. To reduce the formation of primer‐dimers, we selected a concentration of 40 nm for each primer pair. The PCR protocol was adapted from a previous study (Ståhlberg *et al*., 2017). In brief, after an initial denaturation step (3 min, 98 °C), 18 PCR cycles were run with the following settings: 10 s of denaturation at 98 °C, 6 min of annealing at 62 °C, and 30 s of elongation at 72 °C. In the end, a 10‐min elongation step at 72 °C was included. The PCR products were semi‐automatically purified with a Biomek FXP (Beckman Coulter) using AMPure XP beads (Beckman Coulter, Munich, Germany) with a bead‐to‐sample ratio of 0.76 and eluted in 25 μL of nuclease‐free water. By this step, fragments larger than 120 bp were separated from primer‐dimers. In the second PCR step, the same settings were applied except for the following modifications: First, we used 15 μL of DNA products from the first PCR step as input. Second, primers consisting of Illumina universal adapter sequence and a unique combination of the TruSeq DNA HT indexes (forward primer: AATGATACGGCGACCACCGAGATCTACAC‐(Index)‐ACACTCTTTCCCTACACGACGCTCTTCCGATCT; reverse primer: CAAGCAGAAGACGGCATACGAGAT‐(Index)‐GTGACTGGAGTTCAGACGTGTGCTCTTCCGATC) were used at a concentration of 300 nm. Third, the annealing temperature was increased to 72 °C and the annealing time was reduced to 15 s. After the second PCR step, all six multiplexed PCRs of each patient sample were pooled. Ninety micro litre of the mixed PCR products was then cleaned with AMPure XP beads using a bead‐to‐sample ratio of 0.67 and eluted in 20 μL of nuclease‐free water.

### 2.5 Amplicon sequencing

All patient samples were pooled to a single library with a final concentration of 4 nm. The library was sequenced on an Illumina MiSeq using the MiSeq Reagent Kit V2 (Illumina Inc., San Diego, CA, USA) (150 bp single‐end sequencing) with 5% PhiX as spike‐in. Between 30 and 50 patient samples were sequenced in one MiSeq run.

### 2.6 Analysis of sequencing data and variant calling

First, a quality report was generated for each sequenced sample using ‘fastqc’ (Andrew, 2010). Quality reports were examined manually to ensure sufficient sample quality. Subsequently, the ‘trimmomatic’ software version 0.36 (Bolger *et al*., 2014) was used to remove sequenced base pairs with a quality score of < 15 (phred33) at both ends of the reads to avoid false‐positive mutations due to sequencing errors. In addition, a sliding window trimming was performed cutting the read once the average quality within a window of size 4 bp was detected to be < 15. Reads with a length of < 70 base pairs were removed. Next, reads were mapped to the human genome GRCh38 using the ‘bwa mem’ algorithm of the ‘bwa’ alignment software version 0.7.15‐r1140 (Li and Durbin, 2010) with default parameters. ‘samtools’ version 1.4 (Li *et al*., 2009) was used to remove reads that could not be mapped to genome. In addition, reads mapped with a quality of < 13 were excluded. To determine variants, the ‘mpileup’ algorithm implemented in the ‘samtools’ software was applied. The resulting variant calls were summarized and quantified using ‘bam‐readcount’ (https://github.com/genome/bam-readcount), counting only variants at positions with a sequencing quality of at least 30.

### 2.7 Analysis of sequencing variants

The allele frequency was determined for each detected variant by dividing the number of reads containing the variant by the total number of reads mapping to that position. Subsequently, variants were mapped to the amplicon panel. To this end, the genomic coordinates were determined for each amplicon using the ‘BLASTn’ algorithm as implemented in the ‘blast+’ software package (Altschul *et al*., 1990). Here, each amplicon sequence was mapped to the human genome GRCh38 requiring 100% sequence identity. Sequencing variants were then matched to the custom amplicon panel by genomic coordinates. Correctness of the variant mapping and matching was assessed by taking advantage of prior knowledge about mutations in the *NRAS*,* KRAS,* and *BRAF* oncogenes that had previously been determined using Sanger sequencing. In order to distinguish true mutations from false‐positive mutations introduced by, for example, PCR or sequencing errors, a model‐based approach was applied. Assuming that (a) the majority of variants are caused by PCR or sequencing errors, that (b) PCR errors do on average occur at the same frequency at each position of an amplicon, and that (c) sequencing errors at a specific genomic position are equally likely in each sample. A robust linear model of the form

$$\log\left(\frac{a}{1-a}\right)=\beta_{0}+\beta_{1}X_{1}+\beta_{2}X_{2}+\epsilon ,$$

was fit for each amplicon, where *a* is the allele frequency of a variant, *X*_1_ is the variant's position on the amplicon, and *X*_2_ represents the sequenced patient sample. The resulting fit represents a noise model. A variant was considered a true mutation if its model residual exceeded the median of all residuals by at least three standard deviations, indicating that its presence cannot be explained by the estimated noise present in the data. In addition, we required a minimum allele frequency of at least 0.5% for a variant to be considered a true clinically relevant mutation. Next, the remaining mutations were annotated using the COSMIC database of somatic mutations in cancer (Forbes *et al*., 2015). Mutations that were not listed in COSMIC were excluded from further analysis. In addition, COSMIC mutations marked as SNPs were excluded.

### 2.8 Statistics

All *P*‐values reported in this study were computed using a two‐sample Wilcoxon rank sum test as implemented in the r statistical programming language (R Core Team, 2016). As metrics for the relationships between quantitative variables, both the parametric Pearson correlation coefficient and the rank‐based Spearman correlation coefficient are reported in all cases.

### 2.9 Data and software availability

Documented computer code to reproduce analyses presented in this study is available from GitHub at https://github.com/boutroslab/Supplemental-Material/tree/master/Herrmann%26Zhan%26Betge%26Rauscher_2018.

## 3 Results

### 3.1 cfDNA levels correlate with clinical parameters in CRC patients

**Figure 1:**
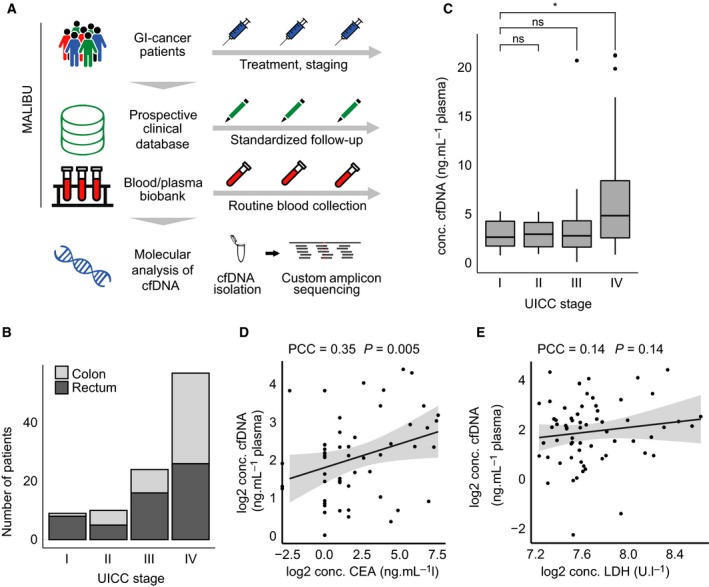
Standardized collection of blood plasma and clinical data from patients with colorectal cancer reveals stage‐dependent levels of cell‐free DNA (cfDNA). (A) The Mannheim Liquid Biopsy Unit (MALIBU) prospectively collects blood and plasma, as well as clinical data from patients with gastrointestinal tumors. Blood is collected at regular time intervals during the course of treatment and follow‐up. The plasma is used for isolation of cfDNA and subsequently for custom amplicon sequencing. (B) Clinical characteristics (location and UICC/AJCC stage) of patients with colorectal cancer were included in the MALIBU biobank and database. (C) Patients with stage IV colorectal cancer have significantly higher cfDNA levels than nonmetastasized patients. cfDNA was isolated from 1‐4 mL plasma of each patient and quantified with qubit. * p < 0.05, two‐sided *t*‐test. (D, E) Association of cfDNA levels with levels of tumor markers. cfDNA levels are significantly associated with CEA levels (*P* = 0.005) (D), but not with LDH levels (E).

To study the value of cfDNA as a biomarker in gastrointestinal cancers, we prospectively collected serial blood samples from cancer patients at a tertiary university hospital in Germany (Mannheim Liquid Biopsy Unit—MALIBU, University Hospital Mannheim, Heidelberg University) (Fig. 1A). For patients undergoing palliative chemotherapy, we obtained blood samples prior to start of treatment and in parallel to each radiologic assessment of therapy response. In all cases, blood sampling was performed before administration of anticancer drugs. For patients undergoing surgical removal of the cancer, we collected blood samples prior and at multiple time points after resection. In parallel, we documented relevant clinical parameters and blood markers in a prospective database. Plasma from each blood sample was isolated within 16 h after sampling and stored at −80 °C until further use. Most samples stored in the biobank were derived from patients with CRC. In total, we collected blood samples from 104 CRC patients across all UICC stages, but predominantly from patients with metastatic CRC (Fig. 1B). To correlate levels of cfDNA with clinical parameters, we measured cfDNA concentrations from blood samples of all CRC patients. Comparison of cfDNA levels demonstrated significantly elevated levels in patients with metastatic compared to nonmetastatic tumors (Fig. 1C). In contrast, no significant difference was found between cfDNA levels of patients with UICC stage I to III cancers. Next, we analyzed the correlation of cfDNA levels with two biomarkers commonly used in gastrointestinal oncology, carcinoembryonic antigen (CEA), and lactate dehydrogenase (LDH) (Fig. 1D,E). We observed a weak, but significant (*P* = 0.005; Pearson's correlation test) correlation of cfDNA concentration with CEA levels. In contrast, no correlation was found with LDH levels. This finding suggests that the cfDNA concentration may be a biological marker that occurs independently of tumor cell necrosis.

### 3.2 A pipeline for the design and analysis of custom amplicon panels for cfDNA

**Figure 2:**
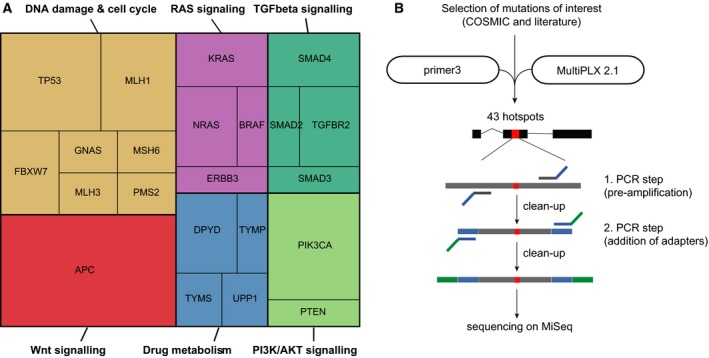
Detection of recurrent mutations in cfDNA with a custom amplicon sequencing workflow. (A) Overview of genes included in the custom amplicon panel. A total number of 43 amplicons were designed to detect hotspot mutations in 18 genes frequently altered in colorectal cancer, including genes related to DNA damage, cell cycle, RAS signaling, TGF beta signaling, Wnt signaling, PI3K/AKT signaling, as well as four genes related to drug metabolism. (B) Experimental workflow of custom amplicon sequencing. Mutational hotspots of interest were selected in COSMIC database and literature, amplicon primers were then designed with Primer3, and multiplexes were determined with multiplx 2.1. A two‐step nested PCR workflow was established, including pre‐amplification and addition of sequencing adapters before PCR clean‐up and subsequent sequencing on MiSeq (Illumina).

**Figure 3:**
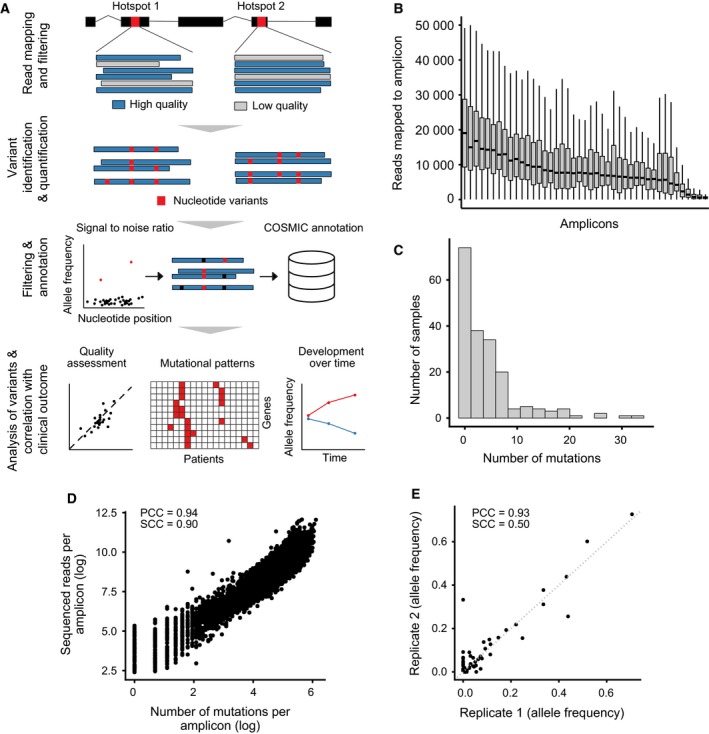
An automated workflow for bioinformatic analysis of amplicon sequencing data from cfDNA. (A) Bioinformatic analysis workflow. Reads generated with Illumina MiSeq were mapped to the human reference genome, and low‐quality reads were excluded before variant identification by comparison of reads and reference. Allele frequency was calculated, and variants were filtered by signal‐to‐noise analysis. Subsequently, variants were annotated with metadata from public databases. Importantly, variants not present in the COSMIC database were filtered out. With this set of mutations, further analyses regarding mutational patterns or the development of mutations over time were performed. (B) Distribution of reads on amplicons. The number of reads mapped to each amplicon region is shown for all 43 amplicons. Data from all cfDNA samples were used for this analysis. (C) Number of mutations per sample. Only mutations with positive signal‐to‐noise ratio and present in COSMIC database were considered true somatic mutations. (D) Association between number of mutations found per amplicon and sequencing depth. Raw mutations were used for this analysis without any filter steps. (E) Correlation of replicates. Two independent replicates were analyzed, starting from cfDNA isolation from the plasma. Shown are the allele frequencies of detected mutations in replicate 1 vs. replicates 2 of 19 analyzed patients.

To analyze mutational patterns in cfDNA, we developed a pipeline for the design of custom amplicon panels. We selected primers that cover mutational hotspots in oncogenes and tumor suppressors of CRC based on the COSMIC database (Forbes *et al*., 2015). In addition, we included primers that amplify frequently mutated loci in genes that affect metabolism of 5‐fluorouracil (Jennings *et al*., 2012; Ooyama *et al*., 2007; Pullarkat *et al*., 2001) or DNA mismatch repair (Li, 2008). These mutations occur at a lower frequency and are not commonly covered by commercial amplicon panels. An overview of all genes can be found in Fig. 2A. We then designed primer pairs that bind 50–75 bp up‐ and downstream of the mutations of interest and calculated optimal multiplexes (see Section 2 for details). Our custom panel contained a total of 43 primer pairs, which were distributed to six multiplex reactions containing 5–9 primer pairs each (see Table S1 for primer sequences and associated mutations). We tested different amounts of cfDNA as input and found that 500 pg per multiplex reaction was sufficient for our nested PCR protocol (see overview in Fig. 2B and Section 2). The purified and pooled amplicons were sequenced on an Illumina MiSeq by 150 bp single‐end sequencing. In parallel, we developed a custom bioinformatic pipeline for the analysis of sequencing results (Fig. 3A). This pipeline comprises four consecutive steps. First, in the quality control step, reads with low sequencing quality are discarded. In the second step, all nucleotide variants across all reads of the same amplicon are detected and quantified. To discriminate PCR or sequencing artifacts from true genetic variants, we developed a robust linear regression model that we applied to each individual amplicon. Alterations identified as true variants by the model were then matched to the COSMIC database and discarded if not covered. By this bioinformatic approach, we assured that only highly confident alterations previously found in cancer tissues are reported.

### 3.3 Functional validation of the amplicon sequencing assay

We tested our amplicon panel in genomic DNA of cancer tissue and cell lines, as well as cfDNA of patients with CRC. First, we evaluated the performance of individual amplicons with our panel. Mapping of sequencing reads to the amplicons showed a continuous distribution of sequencing depth between amplicons (median 7576, minimum 503, maximum 20 809) (Fig. 3B). We found that the number of mutations identified varied between the different samples (Fig. 3C). Also, we noticed a correlation between number of sequenced reads and number of mutations per amplicon (Fig. 3D). Next, we tested the sensitivity of our method to detect specific alterations. To this end, we mixed sheared genomic DNA (fragment size 200–300 bp) from two CRC cell lines (HT29, HCT116) in different ratios. Both cell lines have distinct, monoallelic genetic alterations that can be detected with our amplicon panel. As shown in Fig. S1A, we could reliably identify mutant alleles of HCT116 at a concentration of 1.25% in the total genomic DNA mix, which corresponds to a mutant allele frequency of approximately 0.6%.

We then used our amplicon panel on genomic DNA from formalin‐fixed paraffin‐embedded primary CRC tissues (Fig. S1B). We could detect all RAS mutations previously found by Sanger sequencing, except for two KRAS mutations in Exon 4, which were not covered by the panel. Finally, we tested the reproducibility of our experimental and analysis pipeline by performing amplicon sequencing on independent biological replicates of 19 cfDNA samples, starting from cfDNA isolation from plasma. We show that mutations that occurred at an allele frequency > 0.5% could be detected as indicated by a strong correlation between mutant allele frequencies of the same genetic alterations from independent replicates (Fig. 3E). In summary, our quality control experiments indicate that our custom amplicon sequencing method is functional in both genomic and cfDNA. Furthermore, we identified thresholds for mutant allele frequencies that enable the detection of genetic alterations with high confidence.

### 3.4 Mutational patterns in cfDNA are patient‐specific and highly recurrent

**Figure 4:**
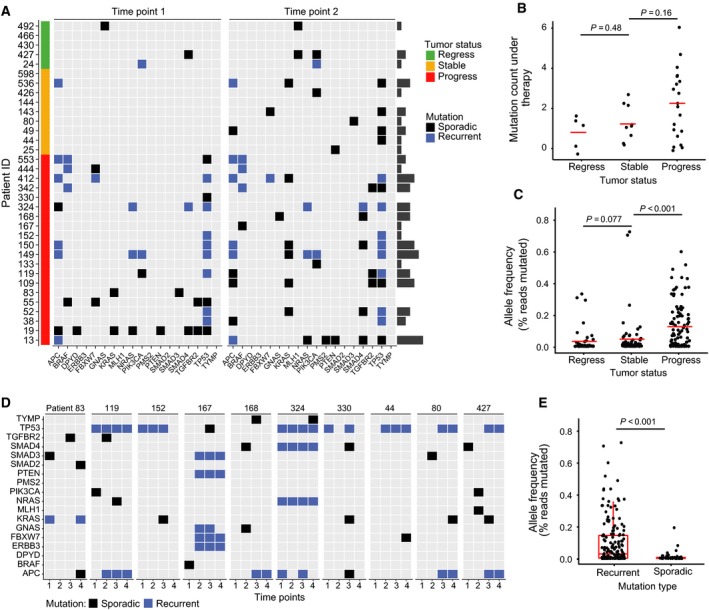
Mutational patterns in cfDNA are patient‐specific and highly recurrent. (A) Heatmap of all mutations detected in cfDNA at two subsequent time points during the course of treatment. Patients’ disease was categorized as regressing, stable, or progressive according to radiologic imaging between the two time points assessed, several mutations were recurrent in both samples, while others were only noted in either time point 1 or 2 (sporadic). (B) Mutation count under therapy. Tumor status was categorized as regressive, stable, or progressive by radiologic imaging at the time the sample was taken. (C) Mutant allele frequency of detected alterations according to tumor status. Tumor status was categorized as regressive, stable, or progressive by radiologic imaging at the time the cfDNA sample was taken. (D) Development of mutational patterns during the course of treatment. Four cfDNA samples were taken from each patient at different time points during the course of treatment, and mutations were analyzed. Several mutations were reoccurring between different time points, while others appeared only at one time point (sporadic). (E) Comparison of mutant allele frequencies of recurrent and sporadic mutations.

Next, we used our custom amplicon panel to analyze mutational patterns in a cohort of 34 patients with advanced CRC, for which serial plasma samples were available. The patients received a chemotherapy backbone consisting of 5‐fluorouracil with or without oxaliplatin or irinotecan. In addition, most patients also received treatment with antibodies targeting Vascular Endothelial Growth Factor (VEGF) or Epidermal Growth Factor Receptor (EGFR). Detailed patient characteristics can be found in Table 1. Within the cohort, 14 patients had a radiologic stable disease or partial remission while the others had disease progression between two consecutive blood sampling time points. Amplicon sequencing of cfDNA demonstrated that 2.41 alterations could be detected on average per sample (Fig. 4A). These alterations were either recurrent (present in serial samples) or sporadic. Alterations occurred at an allele frequency between 0.5% and 96.7% (median 2.25%). Most mutations were found in amplicons of TP53. Overall, we observed a trend toward a higher number of alterations in patients with disease progression (Fig. 4B). Also, we found that mutant allele frequencies were significantly higher (*P* < 0.001) in patients with progressive disease compared to those with stable disease or radiologic therapy response (Fig. 4C), consistent with previous findings (Forshew *et al*., 2012; Gray *et al*., 2015; Newman *et al*., 2014). Using APC as an example case, we observed that the distribution of mutations in cfDNA is very similar to the mutational spectrum found in primary CRC tissue (Fig. S2) (Forbes *et al*., 2017).

**Table 1:** Patient, treatment, and tumor baseline characteristics.^a^. 5‐FU, 5‐fluorouracil or capecitabine; FOLFOX, 5‐FU + oxaliplatin; CAPOX, capecitabine + oxaliplatin; FOLFIRI, 5‐FU + irinotecan; FOLFIRINOX, 5‐FU + oxaliplatin + irinotecan

Parameter	All CRC, *n* = 104	mCRC cohort, *n* = 34
Gender		
Male	74 (71%)	25 (74%)
Female	30 (29%)	9 (26%)
Age (years)	65 (34–88)	65 (36–88)
Stage		
I	9 (9%)	
II	10 (10%)	
III	25 (24%)	3 (9%)^b^
IV	60 (58%)	31 (91%)
Tumor location		
Colon cancer	45 (43%)	15 (44%)
Rectal cancer	58 (56%)	19 (56%)
Both entities	1 (1%)	
Therapy setting		
Surveillance	24 (23%)	
Adjuvant/neoadjuvant	21 (20%)	
Palliative	59 (57%)	34 (100%)
Metastases (only stage IV)		
Hepatic	47 (78%)	24 (71%)
Pulmonary	28 (47%)	18 (53%)
Peritoneal carcinomatosis	14 (23%)	4 (12%)
Pleural carcinomatosis	5 (8%)	2 (6%)
Osseous	4 (7%)	1 (3%)
Other	5 (8%)	0 (0%)
Therapy regimen		
5‐FU	17 (16%)	6 (18%)
FOLFOX/CAPOX	18 (17%)	4 (12%)
FOLFIRI	37 (36%)	22 (65%)
FOLFOXIRI	3 (3%)	2 (6%)
Irinotecan	2 (2%)	0 (0%)
No chemotherapy	25 (24%)	0 (0%)
With anti‐VEGF‐antibody	25 (24%)	16 (47%)
With anti‐EGFR‐antibody	27 (26%)	13 (38%)

^a^ *n* is shown for categorical variables with percentage in parentheses. For continuous variables, median is shown with range in parentheses. ^b ^These three patients all had an inoperable relapse of their primaries.

For a small group of patients, we were able to obtain up to four consecutive blood samples during the course of the therapy, corresponding to a treatment period of approximately 1 year. For all patients in this cohort, we detected both recurrent and sporadic mutations (Fig. 4D). Recurrent mutations were more frequent than sporadic mutations and showed a higher mutant allele frequency (Fig. 4E). Furthermore, we found the pattern of recurrent mutations to be distinct for each patient and conserved throughout multiple blood samples.

### 3.5 Liquid biopsy allows monitoring the clinical course of CRC patients and reveals resistance mechanisms

**Figure 5:**
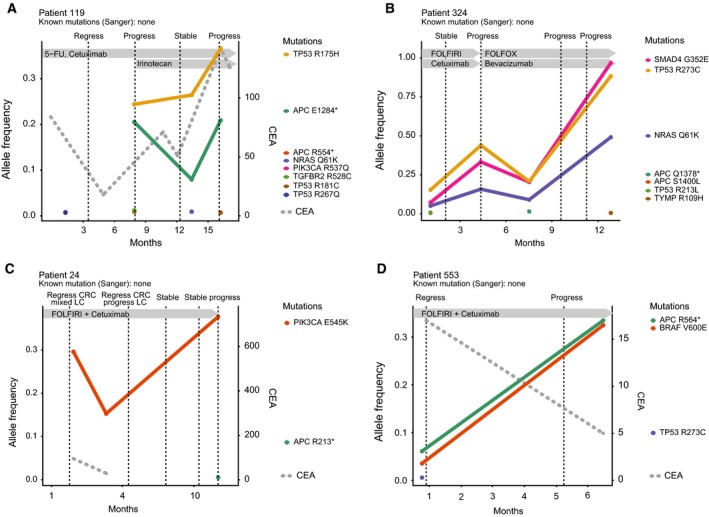
Amplicon sequencing of cfDNA allows monitoring the clinical course of colorectal cancer patients. (A) Patient 119 had received FOLFIRI and cetuximab for metastasized rectal cancer. The patient was in a stable situation and underwent maintenance treatment with 5‐FU and cetuximab by the time of the first liquid biopsy. When the patient experienced radiologic disease progression, treatment was re‐escalated to FOLFIRI and cetuximab, which led to stable disease in the next restaging and progressive disease another 3 months later. Accordingly, novel mutations were discovered by the time of first progression that decreased in allele frequency during stable disease and increased upon radiologic disease progression. (B) Patient 324 had a local recurrence of rectal cancer and hepatic metastases. Under palliative combination treatment with FOLFIRI and cetuximab, the patient had a stable disease by the time of liquid biopsy 1. When he experienced disease progression, liquid biopsy 2 showed increased allele frequencies of several mutations, including an NRAS mutation that had not been detected by Sanger sequencing of the primary tumor. The treatment was switched to FOLFOX + bevacizumab, leading to a decrease in allele frequency, before another radiologic disease progression and, accordingly, rising allele frequencies were observed. CEA levels were not available for this patient. (C) Patient 24 had both a colon cancer with hepatic metastases and a synchronous non‐small‐cell lung cancer. Each tumor manifestation was histologically proven. The colorectal cancer was RAS wild‐type according to Sanger sequencing, and the lung cancer was RAS‐mutated. Nevertheless, the patient received FOLFIRI and cetuximab. During the course of treatment, a PIK3CA mutation that was also identified by amplicon sequencing in material from the colon cancer sank in allele frequency before radiologic imaging proved regression of the colorectal cancer (and progression of the lung cancer). Upon progress of the colon cancer, the mutant allele frequency increased accordingly. (D) Patient 553 was treated for metachronous liver metastases of RAS/RAF wild‐type rectal cancer with FOLFIRI and cetuximab. After initial tumor regression, allele frequency of BRAF V600E and APC R564* mutations rose significantly after radiologic tumor progression, while other mutations, likely from a different tumor subclone, remained on low levels.

Next, we aimed to analyze how the spectrum of mutations found in serial cfDNA samples corresponds to the course of treatment in CRC patients. To this end, we correlated the clinical course with the mutant allele frequency of specific alterations in cfDNA (Fig. 5). All four presented cases were initially treated with 5‐fluorouracil, folinic acid, irinotecan (FOLFIRI), and cetuximab and were in stable disease or had tumor remissions by the time of the first liquid biopsy. Patient 119 had achieved tumor remission and therefore received maintenance treatment with 5‐FU and cetuximab. Two novel mutations occurred in the cfDNA upon radiologic disease progression. The allele frequencies of those mutations closely matched the radiologic extent of disease during the following course of treatment, as did the CEA levels (Fig. 5A). Accordingly, in patients 324 (Fig. 5B), 24 (Fig. 5C), and 553 (Fig. 5D), allele frequencies increased upon tumor progression, while regression or stable disease was associated with stable or decreasing allele frequencies. Interestingly, liquid biopsies revealed an NRAS mutation (patient 324) and a BRAF mutation (patient 553) not identified by Sanger sequencing in the patients’ primary tumors. CEA levels were not congruent with radiologic disease progression and allele frequencies of mutations in cfDNA in patient 553. Hence, in all presented cases, we observed that an increase in allele frequency is closely associated with radiologic progression of the disease. Furthermore, and in contrast to the traditional tumor marker CEA, our amplicon sequencing assay for cfDNA analysis could also reveal biological insights into the mechanisms of therapy resistance to targeted therapies.

## 4 Discussion

In the present study, we describe a flexible experimental and bioinformatic method to analyze custom‐defined gene loci in cfDNA by multiplexed amplicon sequencing. We applied our method on serial cfDNA samples from a pilot cohort of 34 CRC patients to detect recurrent and patient‐specific mutational patterns in key cancer genes.

Over the past decade, significant progress has been made in the analysis of cfDNA from cancer patients. Two recent studies have comprehensively dissected the mutational landscape of cfDNA in large cohorts of patients with CRC (Strickler *et al*., 2018) and other advanced tumors (Zill *et al*., 2018). Both studies demonstrate that the composition of mutations is highly similar between cfDNA and the corresponding primary tumors. Therefore, detection and analysis of cfDNA have been proposed as a biomarker to monitor tumor progression, treatment response, and disease recurrence (Bettegowda *et al*., 2014; Newman *et al*., 2014; Tie *et al*., 2016). Until now, many different approaches have been developed to characterize cfDNA, including the detection of specific mutations (Haselmann *et al*., 2018) or the sequencing of a broad panel of genes (Goodall *et al*., 2017; Quigley *et al*., 2017). Technologies based on both approaches have been approved by the FDA, that is Guardant360 or cobas EGFR Mutation Test V2. A major limitation to their broad clinical application is the diversity of the genetic landscape of cancers from different tissues. Particularly, mutations that occur only in selected cancer types and at medium frequencies can represent potentially druggable conditions, but are not covered by commercial gene panels. Examples include mutations in RNF43 (Steinhart *et al*., 2017) or PMS2 (Khagi *et al*., 2017), which could mark clonal subtypes that may make the tumor responsive to specific agents, such as checkpoint inhibitors. Hence, a main challenge is to customize the analysis of cfDNA to specific tumor types or defined sets of mutations for exploratory analysis. In this regard, our study provides a methodological framework that enables the design and bioinformatic analysis of custom amplicon panels using open‐source software tools. We used our method to design a gene panel tailored for colorectal cancer, including amplicons that cover hotspot mutation sites in oncogenes and tumor suppressor genes, as well as rare mutations in genes relevant for drug metabolism.

We used our custom amplicon panel to analyze sequential cfDNA samples of a pilot cohort of patients with advanced CRC. In accordance with previous studies, we found that most genetic alterations could be found in genes that are known to be highly mutated, such as *APC* or *TP53*. Furthermore, we demonstrate that alterations in specific genes, such as BRAF or KRAS (Diaz *et al*., 2012), occur *de novo* in cfDNA during treatment failure, thereby revealing genetic alterations that can potentially mediate drug resistance. In addition, our data show that the mutant allele frequency correlates with disease progression. Interestingly, we observed that the occurrence of individual mutations remained stable across the course of the therapy. These distinct mutations could be part of a stem mutational signature which is conserved across cfDNA, primary tumors, and metastasis and has been described for a case of breast cancer (Murtaza *et al*., 2015). Our results indicate that such a signature can be found in many patients with CRC. This application might be of particular interest for patients who do not have elevated tumor markers in spite of a high disease burden, which in case of CRC accounts for 30% of patients (Liu *et al*., 2014). However, we are aware that our amplicon sequencing‐based method works best in gastrointestinal cancers that have a limited set of frequently mutated genetic loci, as it is the case for CRC or pancreatic adenocarcinoma. For these cancer types, compact amplicon panels can be designed that cover most hotspot positions. For other cancers, such as gastric or hepatocellular cancer, the mutational load is more equally distributed to many loci, which requires the design of larger amplicon panels for sufficient coverage. This will, however, lead to an increase in cost and technical complexity. An additional goal of our custom amplicon panel was the detection of genetic alterations in genes that are not commonly covered by commercial panels. These include genes involved in 5‐fluorouracil metabolism or regulators of DNA repair. Analysis of cfDNA in our cohort shows that mutations in these genes are rarely found in cfDNA. However, we could detect the *de novo* occurrence of a mutation leading to a premature stop codon in *PMS2* and novel mutations in *TYMP* and *DYPD*. The functional relevance of these mutations is currently unknown. Therefore, a major challenge for the future is the functional annotation of these rare mutations, in order to understand their impact on tumor biology and drug resistance. These insights will enable the profound interpretation of sequencing results from cfDNA in cancer patients.

Although our method enables the flexible design of custom amplicon panels, it still has several limitations. First, calculation of mutant allele frequencies is biased by the presence of genomic DNA from nontumorous cells, which can be increased by unwanted cell lysis. To minimize the extent of contamination by genomic DNA, we selected storage and sample processing conditions that prevent cell lysis, based on findings from other studies (Norton *et al*., 2013; Risberg *et al*., 2018; Wong *et al*., 2013). However, since the time to sample processing varied between 1 and 16 h in our cohort, we are unable to exclude that the measured cfDNA concentrations might be slightly biased by the different storage times. Furthermore, since amplicon sequencing is based on pre‐amplification by PCR, results can be influenced by PCR artifacts and sequencing errors. An effective method to reduce these technical errors is the use of unique molecular identifiers (UMI). For the analysis of cfDNA, methods have been developed to introduce UMI during first PCR amplification cycles by using barcoded primers. These approaches, for instance Safe‐SeqS (Kinde *et al*., 2011) or SiMSen‐Seq (Ståhlberg *et al*., 2017), have been shown to suppress PCR errors and enable more precise counting of mutant allele copies. A drawback of using UMIs is that a vastly higher sequencing depth is required to discriminate between different UMI families, which will increase the costs of the method. However, as sequencing costs will further decrease in the near future, the integration of UMIs in our approach will be feasible. Another limitation of our methods is that it is currently unable to detect copy number alterations or gene fusions, which can be analyzed by other methods (Strickler *et al*., 2018; Zill *et al*., 2018) and provide valuable insights into tumor biology (Stover *et al*., 2018; Weiss *et al*., 2017). Lastly, the prediction of primer pairs that will function in multiplex assays is limited with the currently available bioinformatic tools and we had to discard several primer pairs because they did not yield a functional amplicon. In another case, we were unable to differentiate between mutations and PCR errors due to a poly C region within the MSH6 amplicon region. Therefore, in‐depth testing and experimental validation of primer pairs will be necessary when establishing novel custom amplicon panels. In spite of these limitations, we could show by multiple quality control experiments that our method can reliably detect cancer mutations in cfDNA.

## 5 Conclusions

In summary, we describe a flexible method to analyze mutations in cfDNA by custom amplicon panels. We show that this method can be used to detect recurrent mutational patterns as well as *de novo* mutations in cfDNA in a pilot cohort of patients with metastatic CRC, which enables the monitoring of therapy response and development of drug resistance. Finally, we show that our method can be applied to explore novel mutations at genetic loci that are not covered by commercial amplicon panels. The integration of UMI for error suppression and the validation of the method in a larger, clinically defined cohort of cancer patients will be necessary to further improve our workflow.

## Conflict of interest

The authors declare no conflict of interest.

## Author contributions

SH, TZ, and JB established methods and carried out the experiments. SH, TZ, JB, SB, TG, NS, RJ, NH, TG, and R‐DH collected samples and clinical data. BR performed bioinformatic analysis. MPE and MB provided funding and supervised the project. SH, TZ, JB, and MB wrote the manuscript text.

## Supporting information


**Fig. S1.** Sensitivity of the amplicon sequencing assay.
**Fig. S2.** Distribution of APC mutations in primary colorectal cancer and cfDNA.

**Table S1.** List and characteristics of primers in the amplicon panel.

## Data Availability

All sequencing data generated for this study are available from the European Genome‐phenome Archive under the accession number EGAS00001003382. Documented computer code to reproduce the figures of this manuscript is available from GitHub at https://github.com/boutroslab/Supplemental-Material/tree/master/Herrmann%26Zhan%26Betge%26Rauscher_2018.
