# Supplementary material for: Detection of mutational patterns in cell‐free DNA of colorectal cancer by custom amplicon sequencing
Source: Mol Oncol. 2019 Jul 19;13(8):1669–83. doi: 10.1002/1878-0261.12539 (PMC6670011; doi:10.1002/1878-0261.12539)
Supplement: Supplementary file 1 — Fig. S1. Sensitivity of the amplicon sequencing assay. Fig. S2. Distribution of APC mutations in primary colorectal cancer and cfDNA. [file MOL2-13-1669-s001.pdf]

SUPPLEMENTARY FIGURES

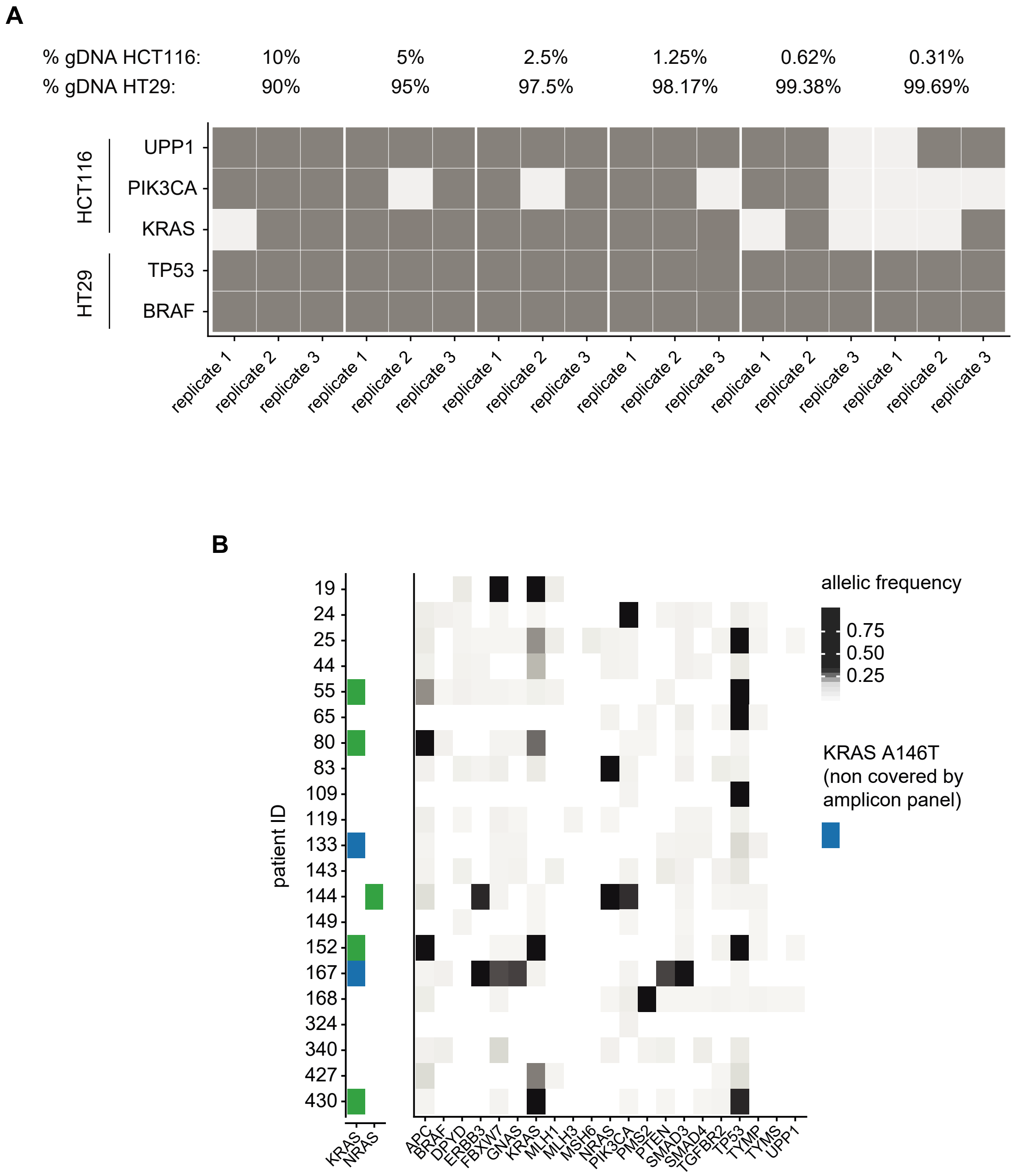

Supplementary Figure 1: Sensitivity of the amplicon sequencing assay.

(**A**) Sheared DNA (200-300bp) isolated from HCT116 cells with known KRAS, PIK3CA and UPP1 mutations were mixed with HT29 cells (with known BRAF and TP53 mutations) in decreasing concentrations. The sequencing assay securely detected mutations down to a fraction of 1,25% HCT116 DNA (i.e. an allele frequency of 0.0062).

(B) Analysis of primary tumor samples. DNA was isolated from formalin-fixed paraffin-embedded tissue sections after macrodissection to enrich for tumor cell content. Amplicon sequencing was performed as for liquid biopsy samples. Sanger sequencing for hot-spot mutations in KRAS and NRAS had been routinely performed with DNA isolated from FFPE during diagnostic work-up of the patients.

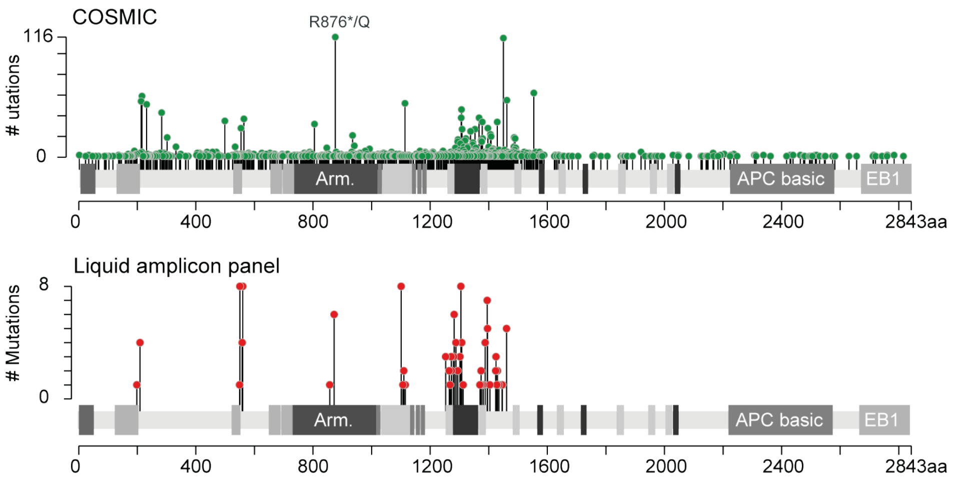

Supplementary Figure 2: Distribution of APC mutations in primary colorectal cancer and cfDNA

Mutations in APC in colorectal cancer in the COSMIC database (upper panel) and mutations in APC detected in cfDNA by our amplicon assay (lower panel).
